# The Prognostic Value of HPV Status and p16 Expression in Patients with Carcinoma of the Anal Canal

**DOI:** 10.1371/journal.pone.0108790

**Published:** 2014-10-01

**Authors:** Gloria B. Roldán Urgoiti, Karla Gustafson, Alexander C. Klimowicz, Stephanie K. Petrillo, Anthony M. Magliocco, Corinne M. Doll

**Affiliations:** 1 Department of Medicine and Oncology, University of Calgary, Calgary, Alberta, Canada; 2 Department of Oncology, Tom Baker Cancer Centre, Calgary, Alberta, Canada; 3 Department of Anatomic Pathology, H. Lee Moffitt Cancer Center, Tampa, Florida, United States of America; Georgetown University, United States of America

## Abstract

**Background:**

In anal cancer studies, the detection frequency of high-risk HPV (human papillomavirus) is variable, depending on the method used. There are limited data reporting results of different HPV detection techniques in the same clinical series, and very few correlating results with clinical outcome.

**Objectives:**

To evaluate tumor expression of p16/HPV16 using three different methods, and to determine their association with clinical outcome in patients with anal canal squamous cell carcinomas (SCC).

**Design:**

This retrospective study included patients with anal canal SCC treated with definitive radiotherapy or chemoradiotherapy at a single institution between 1992 and 2005. Formalin-fixed paraffin–embedded tumor samples from 53 of the 89 (60%) patient pre-treatment biopsies were adequate for tissue microarray construction. HPV status was determined using: p16 expression by conventional immunohistochemistry (IHC) and quantitative IHC (AQUA), HPV genotype analysis by chromogenic *in situ* hybridization (CISH) and HPV linear array sub-typing. Expression status was correlated with clinical outcome.

**Results:**

80% (28/35) of patient tumors had high p16 expression using conventional IHC. HPV16 CISH was positive in 81% (34/42) of tumors, and 78% (28/36) of tumors were HPV subtype 16. HPV16 CISH correlated with p16 evaluated by conventional IHC (correlation coefficient 0.46; p = 0.01) and by p16 AQUA score (correlation coefficient 0.49; p = 0.001). A subset of cases (15%) had very high p16 quantitative IHC scores (>244) and were associated with a higher incidence of local or distant recurrence (p = 0.04).

**Conclusions:**

The vast majority (80%) of anal canal SCC in our series were positive for HPV16/p16, regardless of the testing method used. The exploratory analysis of automated quantitative IHC scoring was the only technique to define a subset of patients with a worse prognosis by p16 expression status on univariate analysis. Further exploration of the molecular mechanisms of treatment resistance in association with very high p16 expression is warranted.

## Introduction

Anal cancer, although a relatively rare malignancy, has been steadily increasing in incidence over the last fifty years [1], [2]. Although chemoradiotherapy (CRT) has become the standard treatment for patients with this disease, there has been little improvement in survival in the last few decades [3], [4]. There is emerging interest in the determination of tumoral molecular factors that may predict for response or resistance to standard CRT.

Recently, there have been multiple studies published on the prognostic significance of human papillomavirus (HPV) status and outcome in patients with squamous head and neck cancers, with improved outcome in patients with HPV-positive versus HPV-negative tumors [5]–[8]. However, the biological basis for this improved outcome in HPV-positive cases is not clear.

The impact of HPV status and HPV pathway activation on prognosis in patients with anal cancer has not been well studied. High risk HPV is a well known etiologic agent in the development of anal cancer, but the frequency of detection is dependent on the diagnostic method utilized. In anal cancer studies, high-risk HPV has been reported in about 22% of cases using immunohistochemistry (IHC) for E6 protein [9], and in up to 90% of cases using PCR [1], [10] and DNA microarrays [11]. P16 expression has been used by different authors to detect HPV-associated tumors specifically in anal canal carcinoma. Lu et al [12] evaluated the expression of p16, Rb and p53 proteins using IHC in 29 cases of anal squamous cell carcinoma, and used PCR to test HPV status. They found that 100% of HPV positive tumors had strong and diffuse staining for p16. In a more recent series of 76 patients with anal carcinoma, p16 was positive by IHC in 92% of the tumors (n = 68 tested) with two p16-positive samples negative for HPV16 by RT-PCR [13].

Although techniques involving direct HPV 16 DNA identification may be considered the “gold standard” for the determination of HPV status in human tumors, these tests are technically challenging and relatively expensive for diagnostic laboratories to perform on a routine basis [14], [15]. P16 protein expression is gaining acceptance as a cost-effective surrogate marker for HPV infection [16]. The diagnostic utility of p16^Ink4a^ overexpression had been demonstrated in high risk HPV infections, including cervical dysplasia and carcinoma [16]–[18] while is actively being evaluated in head and neck tumors [19].

Given the differences in methods for determination of HPV status in patients with squamous cell cancers in the literature, we developed this study to evaluate and compare tumor expression of p16/HPV16 using three different methods and its association with clinical outcome in a cohort of patients with anal cancer, treated with radical radiotherapy (RT) or CRT. This is the first report using these techniques in the evaluation of HPV status in anal cancer patients.

## Materials and Methods

### Patient and treatment characteristics

This retrospective study included patients diagnosed with anal squamous cell cancer (SCC) treated with definitive RT or CRT, with curative intent, at a single institution (Tom Baker Cancer Centre, Calgary, AB, Canada) between 1992–2005. Patients had at least three years of follow-up data and no other active malignancy at the time of diagnosis. Approval for the study was obtained from the University of Calgary Conjoint Health Research Ethics board. Waiver of consent was granted by the Research Ethics board for this study; all patient information was anonymized and de-identified prior to analysis. Eighty-nine patients were identified from the Alberta Cancer Registry using these eligibility criteria, and clinicopathological data were retrieved from the patients' charts. Standard pre-treatment evaluation included physical examination, computed tomography (CT) of the abdomen and pelvis, tumor biopsy, and bloodwork [complete blood count (CBC), electrolytes, liver and renal function tests]. In addition, patients had weekly bloodwork on-treatment, including CBC, electrolytes, urea, and creatinine. After completing treatment, patients were generally followed clinically every three months for the first year, every four months for year two, and every six months to year five. Follow-up imaging was ordered as clinically indicated.

### Tissue microarray (TMA) building

Formalin-fixed paraffin–embedded (FFPE) tumor samples from 53 of the 89 (60%) patient pre-treatment biopsies were adequate for tissue microarray (TMA) construction. After pathologist review (AM) to confirm squamous cell histology and the location of tumor for sampling, TMAs were assembled from triplicate 0.6 mm cores of FFPE primary tumor samples using a Beecher Manual Tissue Arrayer (Beecher Instruments, Sun Prairie, WI, USA).

### p16 immunostaining

Four µm thick sections were cut from the TMA blocks and deparaffinized in xylene, rinsed in ethanol, and rehydrated. Heat induced epitope retrieval was performed by heating slides to 121°C in a citrate-based buffer (pH 6.0) Target Retrieval Solution (Dako, Mississauga, ON, Canada) for 8 minutes in a decloaking chamber (Biocare Medical, Concord, CA, USA). Slides were stained using a Dako autostainer. Endogenous peroxidase activity and unspecific staining for conventional p16 IHC was quenched using a peroxidase block (Dako) for 5 minutes and for fluorescence p16 IHC was quenched with peroxidase block for 10 minutes followed by a 15 minute protein block (Dako). Slides were incubated with a 1∶100 dilution of anti-p16 mouse monoclonal (JC8 clone, Novus Biologicals, Littleton, CO, USA) in antibody diluents (Dako) at room temperature for 30 minutes for conventional IHC and 60 minutes for fluorescence-IHC. Slides were washed with TBST wash buffer (Dako), and goat anti-mouse antibody conjugated to a horseradish peroxidase-decorated dextran polymer backbone from the Dako EnVision+ system was applied for 30 minutes for conventional IHC. Dako Evision+ was applied with 1∶200 dilution of Alexa-555 conjugated goat anti-rabbit antibody (Invitrogen, Burlington, ON, Canada) for 60 minutes at room temperature for fluorescence-IHC. Slides were washed with TBST wash buffer (Dako), and DAB chromogen from the DAKO EnVision TM + system (Dako) was applied for 10 minutes for conventional IHC and TSA-Plus Cy5-tyramide signal amplification reagent (PerkinElmer, Woodbridge, ON, Canada) was applied for 5 minutes for fluorescence-IHC. The slides were washed with TBST wash buffer (Dako). For conventional IHC slides were then incubated for 2 minutes with Mayer's Hematoxylin (Dako) followed by two minutes of incubation with Harleco blueing reagent. Slides were then dehydrated in ethanol baths and rinsed in xylene. Slides were cover slipped and sealed using Cytoseal XYL mounting medium (Richard-Allan Scientific, City). For fluorescence IHC the slides were mounted in Vectashield Mounting Medium with DAPI (Vector Laboratories, Burlington, ON, Canada) and stored at 4°C until use.

### Conventional p16 (diaminobenzydene, DAB) scoring

Slides were evaluated by a pathologist (AM) using light microscopy and scored on a 0–3 scale: 0, complete absence of tumor staining; 1, weak staining of tumor cells; 2, less than 50% tumors cells stained, moderate intensity; 3, greater than 50% tumor staining with moderate to intense staining.

### AQUA p16 analysis

**Figure 1:**
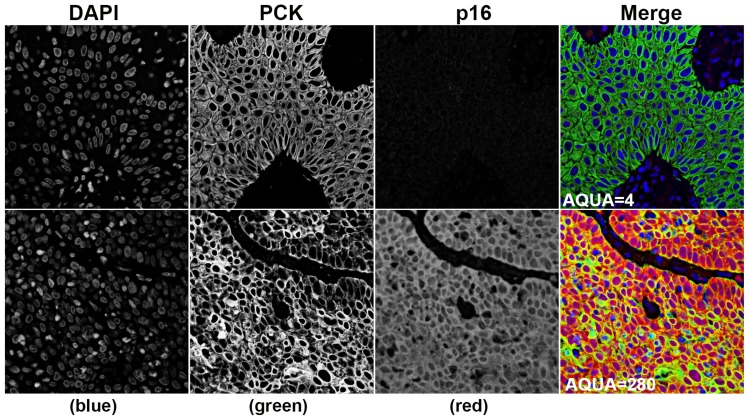
Representative examples of low and high AQUA scores. 4',6-diamidino-2-phenylindole (DAPI) defines the nuclear compartment, Alexa-555 defines pan-cytokeratin (PCK) positive anal carcinoma cells and the tumor cytosolic compartment. The “merge” panels are pseudo-colored red for p16, green for pan-cytokeratin (epithelial cell/tumor marker), and blue for DAPI (nuclear stain).

Automated image acquisition was performed using a HistoRx PM-2000, which has previously been described in detail [20], [21]. Briefly, high resolution monochromatic 8-bit (resulting in 256 discrete intensity values per pixel of an acquired image) digital images were obtained for every histospot on the tissue microarrays using filters specific for 4',6-diamidino-2-phenylindole (DAPI) to define the nuclear compartment, Alexa-555 to define pan-cytokeratin positive cervical carcinoma cells and the tumor cytosolic compartment, and Cy5 to define the target biomarker P16. Pixels were then written to image files as a function of power [Power (P) = (Pixel Intensity/256)/exposure time)] in order to help compensate for experimental variations in staining intensity. Analysis of the digital images was performed with AQUAnalysis software version 2.2.1.7. Briefly, a tumor specific mask was generated to distinguish the carcinoma cells from supporting stromal tissue by thresholding the pan-cytokeratin images. Thresholding created a binary mask that identified the presence or absence of tumor cells by the presence of a pixel that was ‘on’ or ‘off’, respectively. Thresholding levels were verified, and adjusted if necessary, by spot-checking a small sample of images to determine an optimal threshold value. All images were then processed using this optimal threshold value. The PLACE (Pixel-based Local Assignment for Compartmentalization of Expression) algorithm then assigned each pixel in the target images to either the nuclear or cytoplasmic compartment. Once each pixel was assigned to a subcellular compartment, the signal in each location was tabulated and used to generate compartment specific AQUA scores, which reflect the average signal intensity per compartment area. The target p16 signal in the masked areas was tabulated and used to generate tumor-specific AQUA scores, which reflect the average signal intensity per tumor area. Figure 1. Images were validated according to the following: 1)>10% of the tissue area is pan-cytokeratin positive, 2)>50% of the image was usable (i.e. not compromised due to overlapping or out of focus tissue). Unusable areas within each image were manually cropped so that they were excluded from the final analysis.

### HPV CISH technique

For HPV genotype analysis, HPV chromogenic *in situ* hybridization (CISH) analysis was performed on 4 µm sections cut from TMAs. The slides were stained using the Ventana INFORM HPV III Family 6 and 16 probes and the iVIEW Blue+ v3 kit on the Ventana BenchMark staining platform with standard protocols (Ventana Medical Systems, Inc., Tucson, AZ, USA). The low-risk family 6 probe detects HPV genotypes 6 and 11 and the high-risk family 16 probe cocktail detects high-risk HPV genotypes 16, 18, 31, 33, 35, 39, 51, 52, 56, 58, and 66. Established controls, such as the HPV 3-in-1 System Control Slides, were processed and run with all clinical specimens. The CaSki section is a cell line that reportedly contains approximately 200–400 copies of HPV type 16 per cell and the HeLa section is a cell line that reportedly contains approximately 10–50 copies of HPV type 18 per cell. The C-33 A negative section is a cell line, which is an HPV-negative human cervical carcinoma. Slides were evaluated using light microscopy and scored on a 0–3 scale: 0, complete absence of signal in tumor cells; 1, faintly staining individual foci; 2, moderately staining tumor cells with numerous small foci to small clusters, 3, intensely staining clusters within tumor cells.

### HPV linear array sub-typing

HPV sub-typing was completed using the Roche Linear Array genotyping assay (PCR-LA; Roche Molecular Systems, Inc., Branchburg, NJ). Approximately twenty 12 µm slices of each sample were treated twice with 1 mL 100% xylene, and then washed twice with 1 mL 100% ethanol to remove any excess xylene. Open tubes containing the washed tissue were incubated for 15 minutes at 37°C to evaporate residual ethanol. DNA was extracted from the tissue using the Roche AmpiLute Liquid Media Extraction Kit or Qiagen DNeasy Blood and Tissue Kit. Extracted DNA was put through Linear Array DNA PCR protocol using an Eppendorf Mastercycler epGradient thermal cycler according to manufacturer directions as described by Wong et al. [22]. Detected subtypes were manually interpreted qualitatively using the reference guide provided in the Linear Array kit. Positive, negative, and blank controls were run each time to ensure lack of contamination and validity of results. When both high and low internal β-Globin control probe lines were visible on the test strip and no HPV sub-types were visible, samples were reported to be HPV negative.

### Statistical analysis

The expression of each molecular marker was measured in triplicate and the maximum score was used for analysis. This applied for grading scores (i.e. IHC 0–3 score) as well as for AQUA continuous scores. Clinical complete response (CR) was defined as no clinical evidence of tumor on physical examination, and partial response (PR) was defined as a reduction of at least 50% of the clinical size of the primary tumor, determined at three months after completion of RT or CRT. Progression-free survival (PFS) was defined as the interval between diagnosis and evidence of recurrence/progression, death or lost to follow-up. Overall survival (OS) was calculated from date of diagnosis to death or lost to follow-up. X tile.Ink software was used to identify a cutpoint in AQUA continuous scores associated with increased PFS and OS for additional exploratory analysis.

Results were tabulated and analyzed with STATA Version 12.0 and were two-tailed. Non-parametric Spearman's correlation test was used to identify correlations between molecular markers. Chi-square or Fisher's exact tests were used to determine the significance of associations between categorical variables. Clinical and molecular variables were evaluated for association with survival using Kaplan-Meier survival analysis with log-rank statistic for determining significance. Variables that proved to be significant in univariate analysis were selected for Cox multivariate analysis (forward stepwise Wald) to assess their independent predictive value. P-values less than 0.05 were considered statistically significant.

## Results

### Patient, tumor and treatment characteristics

89 patients were identified, of whom 53 (60%) had pre-treatment tumor specimen available for molecular analysis. Details are shown in Table 1. There were no significant differences in patient, tumor or treatment factors between tested and non-tested cases. Chemotherapy consisted of 5-fluorouracil, mitomycin C (MMC), folinic acid. In our center HIV is not a common diagnosis; patients are screened for high risk factors and tested accordingly. One patient was found to be HIV-positive on testing.

**Table 1:** Characteristics of 89 patients with anal canal carcinoma.

	All patients	Not Tested	Tested Patients	
Characteristic	(N = 89)	(N = 36)	(N = 53)	P value
Age (years, mean); (range)	58 (34–86)	58 (36–86)	59 (34–83)	0.7 (t-test)
Gender (male/female)	29/60	16/20	13/40	0.07 (Fisher's)
Smoker (total)	50(80)	17(32)	33(48)	0.3 (Fisher's)
ECOG PS 0	47 (52.8%)	18 (50%)	29 (59%)	0.5 (Fisher's)
ECOG PS 1	26 (29.2%)	14 (39%)	12 (24%)	
ECOG PS 2	10 (11.2%)	3 (8%)	7 (14%)	
ECOG PS 3	2 (2.2%)	1 (2.7%)	1 (2%)	
ECOG PS unknown	4 (4.5%)	0 (0%)	4 (8%)	
Tumor size (cm, mean);(range)	3.8 (0.4–10)	3.6 (0.4–10)	3.9 (1–9.8)	0.5 (t-test)
Tumor size≥4 cm	33/70 (47%)	10/27 (37%)	23/43 (53%)	0.2 (Fisher's)
Nodal status (N): N0	52 (58%)	21 (58%)	31 (58%)	0.7 (Fisher's)
N1	1 (1%)	1 (2.7%)	0 (0%)	
N2	7 (7.9%)	4 (11%)	3 (5.6%)	
N3	5 (5.6%)	2 (5.5%)	3 (5.6%)	
NX	24 (27%)	5 (14%)	16 (30%)	
Median RT dose to tumor (Gy)	52	53	55	0.5 (t-test)
Concurrent chemotherapy	72 (81%)	31 (86%)	41(77%)	0.4 (Fisher's)

ECOG PS: Eastern Cooperative Oncology Group Performance Status; RT: radiotherapy.

At three months post-treatment, clinical complete response was observed in 73% (55/75). There were 24 relapses (27%) in the whole group. Mean time to relapse was 73 months (95% CI 63–82). At a median follow-up of 59 months, forty-six patients (52%) had died. Median overall survival was 82 months (95% CI 61–103).

### P16, HPV 16 and HPV Subtyping

**Figure 2:**
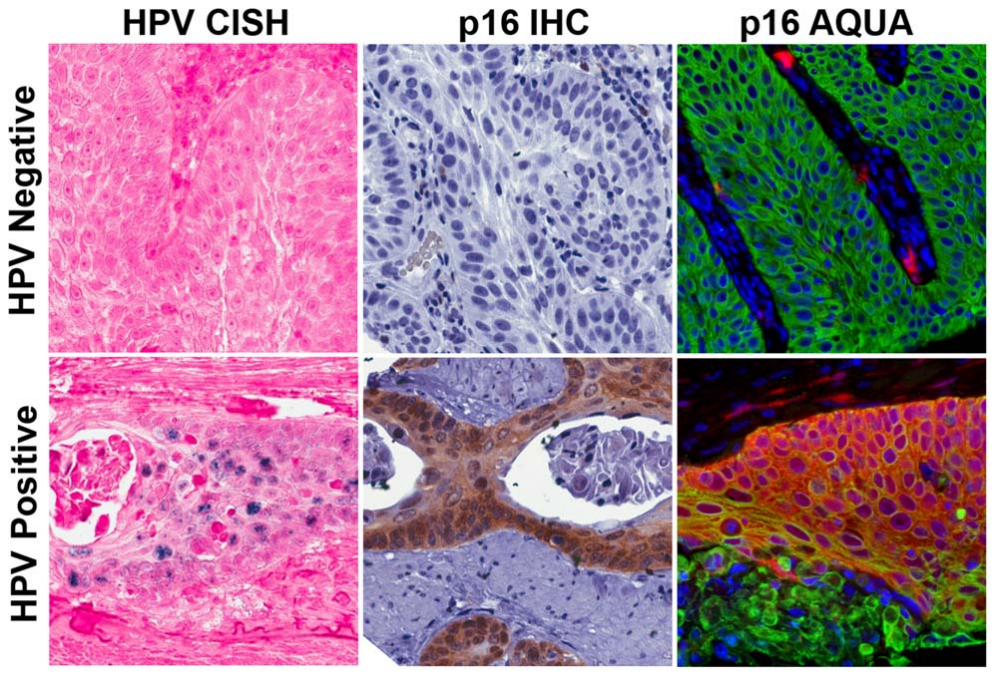
Representative examples of non-serial sections from HPV 16 negative and positive cases by subtyping. HPV 16 negative (top panels) and an HPV 16 positive (bottom panels) cases by subtyping, using HPV CISH (left panels), p16 IHC (middle panels), and p16 AQUA (right panels). P16 AQUA images are pseudo-colored red for p16, green for pan-cytokeratin (epithelial cell/tumor marker), and blue for DAPI (nuclear stain).

Of 53 pre-treatment tumor specimens, molecular testing was performed as follows: conventional p16 (DAB) scores, n = 35; p16 AQUA, n = 46; HPV16 CISH, n = 42; HPV subtyping, n = 36. Thirteen random samples underwent re-testing, with identical results (not shown). Eight of the thirteen re-tested samples were completed on separate DNA extractions from the tissue blocks, and results were also identical in these cases, indicating no inter-extraction variation. Representative “high” and “low” cases using the different methods are shown in Figure 2, in tumors that were either positive or negative by HPV16 subtyping.

**Figure 3:**
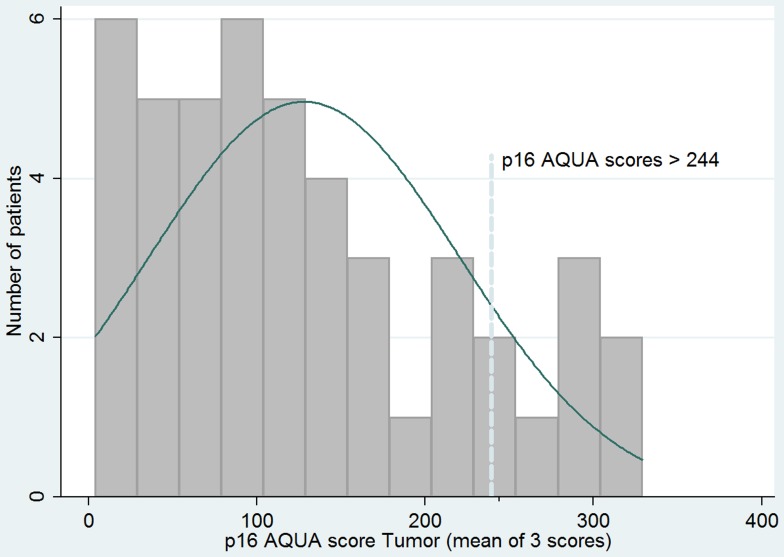
Histogram of AQUA scores with cutpoint identifying very high expression of p16.

Using conventional IHC analysis for p16, 80% (28/35) of patient tumors over-expressed p16 (score 3). Mean p16 tumoral AQUA score was 128 (range 4 to 315); 15% of tumors had AQUA>244, very high on the AQUA score histogram (Figure 3). HPV16 CISH was positive in 81% (34/42) of tumors. Of tumor specimens analyzed for HPV subtype, 78% (28/36) were HPV 16; in 2 cases there was co-expression with HPV18.

### Clinical and molecular factors and outcome, univariate and multivariate analyses

**Figure 4:**
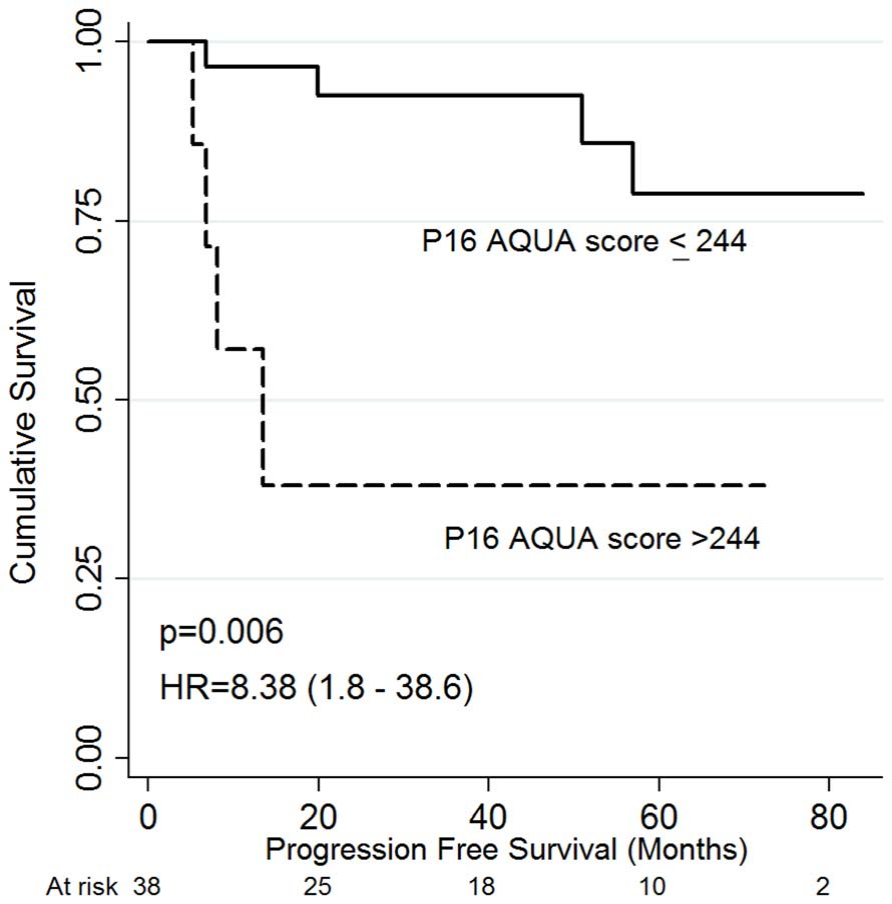
Progression-free survival according to P16 AQUA score. P16 AQUA score with a cutpoint at 244 in patients with pretreatment hemoglobin≥120 g/L.

**Figure 5:**
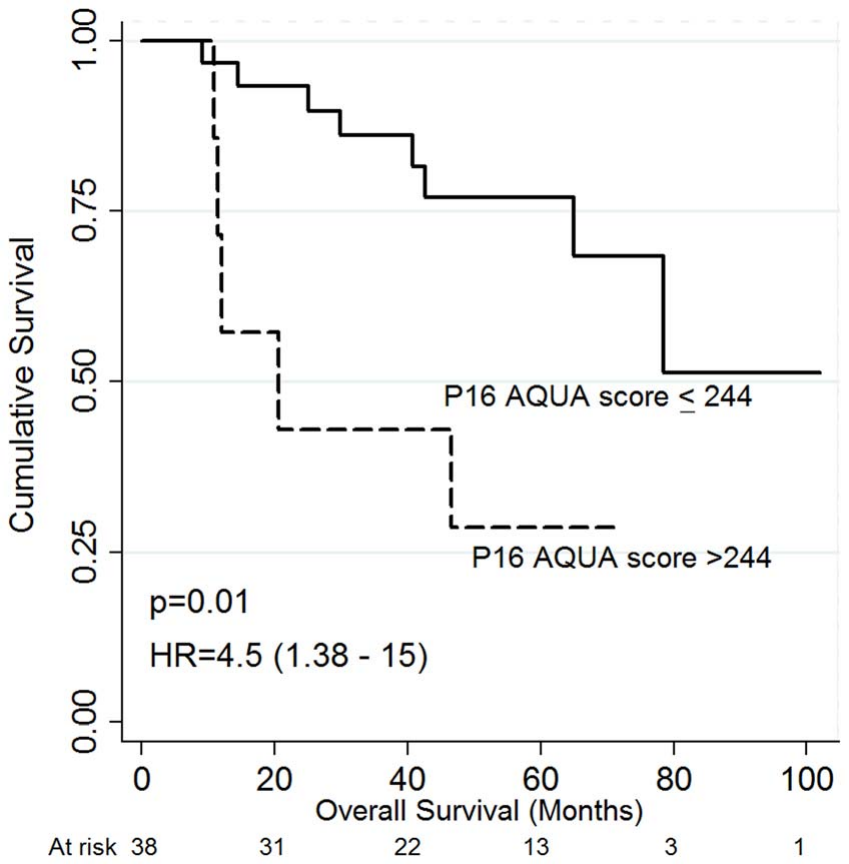
Overall survival according to P16 AQUA score. P16 AQUA score with a cutpoint at 244 in patients with pretreatment hemoglobin≥120 g/L.

Worse 5-year PFS was associated with poor performance status, larger tumor size, lower pre-treatment hemoglobin status (<120 g/L), positive nodal status and very high tumoral p16 status by AQUA (score>244) (Table 2). Notably, very high tumoral p16 status by AQUA was associated with worse PFS and OS in patients with higher pre-treatment hemoglobin (≥120 g/L), [HR 8.4 (95% CI 1.8–38.6), p = 0.006], Figure 4; OS [HR 4.5 (95% CI 1.4–15), p = 0.01], Figure 5. In addition, very high AQUA score status was associated with a higher incidence of local or distant recurrence (p = 0.04). Worse OS was associated with poor performance status, larger tumors, positive nodal status, lower pretreatment hemoglobin, treatment RT alone, and incomplete response at three months post-treatment, on univariate analysis (Table 3). Neither p16 status by conventional IHC nor HPV16 status via CISH was associated with outcome, Tables 2 and 3. On multivariate analysis, worse PFS was associated with poor performance status: [HR 6.7 (95% CI 2.2–20.5). p = 0.001], positive nodal status: [HR 2.1 (95% CI 1.1–3.9), p = 0.021], and incomplete response at three months [HR 2.3 (95% CI 1.03–5.0), p = 0.041. On multivariate analysis, worse OS was associated with poor performance status: [HR 2.8 (95% CI 1.1–6.8), p = 0.02], lower pre-treatment Hb (<120 g/L): [HR 4.8 (95% CI 1.1–21.4), p = 0.04], and incomplete response at three months: [HR 2.6 (95% CI 1.3–5.0), p = 0.007]. P16 AQUA was not independently associated with PFS or OS.

**Table 2:** Univariate analysis: 5-year progression-free survival (PFS).

VARIABLE	5 year PFS HR (95% CI)	P value
Gender (Female/male)	0.47 (0.2–1.05)	0.07
Age	0.98 (0.94–1.01)	0.25
ECOG PS	2.8 (1.9–4.3)	<0.0001
Tumor size ≥4 cm	4.0 (1.4–11.4)	0.009
Nodal status (positive vs negative)	4.24 (1.6–11.3)	0.004
Pretreatment Hb<120 g/L	5.6 (2.2–14.1)	<0.0001
RT (n = 17) vs CRT (n = 72)	2.0 (0.7–5.6)	0.19
Response at three months	0.44 (0.29–0.73)	0.002
P16 DAB score	0.67 (0.34–1.31)	0.24
P16 DAB score 3	0.37 (0.75–1.86)	0.27
HPV16 CISH	1.22 (0.69–2.18)	0.49
P16 AQUA tumor score, quartiles	1st – 0.62 (0.15–2.61)	0.65
	2nd – 0.95 (0.22–4.02)	0.94
	3rd – 0.21 (0.02–1.77)	0.15
	4th – 1.0	
P16 AQUA tumor score>244	3.5 (1.04–11.8)	0.04
P16 AQUA tumor score>244 and higher pretreatment Hb (≥120 g/L)	8.4 (1.8–38.6)	0.006

ECOG PS: Eastern Cooperative Oncology Group Performance Status; RT: radiotherapy; CRT: chemoradiotherapy; Hb: hemoglobin; DAB: diaminobenzydene, CISH: chromogenic *in situ* hybridization

**Table 3:** Univariate analysis: 5-year overall survival (OS).

VARIABLE	5-year OS HR (95% CI)	P value
Gender (female vs male)	0.58 (0.32–1.04)	0.075
Age	1.01 (0.99–1.04)	0.26
ECOG PS	3.2 (2.2–4.6)	<0.0001
Tumor size ≥4 cm	2.8 (1.3–5.9	0.008
Nodal status (positive vs negative)	2.43 (1.09–5.42)	0.029
Pre-treatment Hb<120 g/L	4.5 (2.3–9.1)	<0.0001
RT vs CRT	3.86 (2.03–7.35)	<0.0001
Response at three months	0.35 (0.23–0.53)	<0.0001
P16 DAB score	0.90 (9.49–1.65)	0.75
P16 DAB score 3	0.38 (0.12–1.22)	0.09
HPV16 CISH	1.23 (0.64–2.38)	0.52
P16 AQUA tumor score, quartiles	1st – 0.61 (0.18–2.08)	0.43
	2nd – 1.55 (0.49–4.86)	0.45
	3rd – 0.61 (0.17–2.18)	0.45
	4th – 1.0	
P16 AQUA tumor score>244	2.34 (0.84–6.54)	0.09
P16 AQUA tumor score>244 and higher pretreatment Hb (≥120 g/L)	4.5 (1.4–15)	0.01

ECOG PS: Eastern Cooperative Oncology Group Performance Status; RT: radiotherapy; CRT: chemoradiotherapy; Hb: hemoglobin; DAB: diaminobenzydene; CISH: chromogenic *in situ* hybridization.

### Correlation between molecular markers

**Figure 6:**
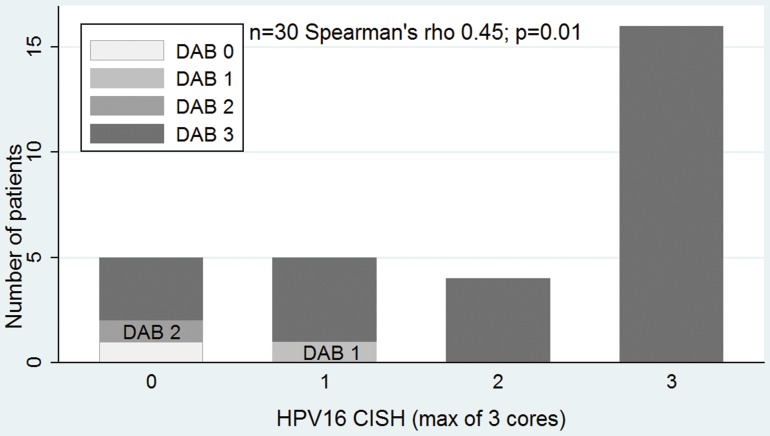
CISH for HPV16 and DAB score correlation. DAB: conventional p16 diaminobenzydene.

**Figure 7:**
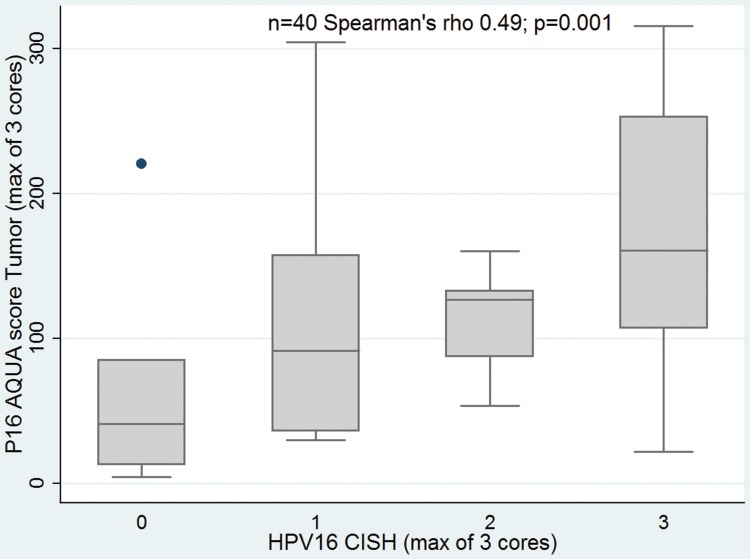
CISH for HPV16 and AQUA score correlation.

**Figure 8:**
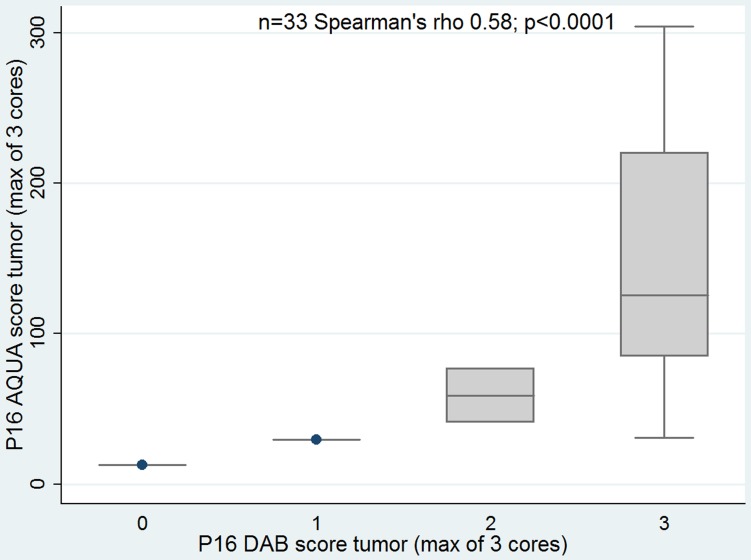
p16 evaluated by DAB and AQUA. DAB: conventional p16 diaminobenzydene.

PV16 CISH correlated with p16 evaluated by conventional IHC DAB score (correlation coefficient 0.46; p = 0.01) and by p16 AQUA score (correlation coefficient 0.49; p = 0.001). Also, there was association of p16 by conventional IHC and AQUA (correlation coefficient 0.58; p<0.0001), Figures 6–8.

**Figure 9:**
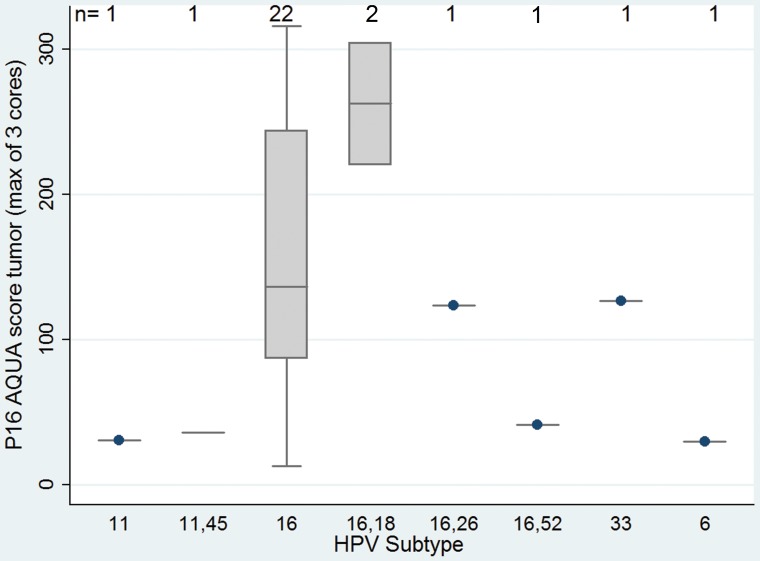
P16 expression by AQUA according to HPV subtype.

When dividing the classified tumors into HPV16 and other subtypes, we found there was correlation with the three applied techniques as follows: p16 DAB (Chi square; p = 0.009); p16 AQUA score (Spearman's; p = 0.02) and tended to correlate with HPV16 by CISH (Spearman's; p = 0.06). The correlation between HPV subtypes and AQUA is showed in Figure 9.

Considering HPV subtyping as the “gold standard” to distinguish HPV16 as positive and negative, and using conventional IHC for p16 from a total of 24 cases analyzed by both methods, there were two false positive cases and one false negative case. In 30 cases both HPV subtyping and CISH analysis were performed. CISH showed four false negative cases and three false positive.

Seven patients (8%) had AQUA p16 scores>244. Six of 7 were HPV16; one additionally co-expressed HPV18. By CISH, p16 was score 3 in 6/7 of these cases and score 1 in the other one. Finally, 5/7 had p16 evaluated by conventional IHC and all of them were graded as score 3.

## Discussion

In this study, we utilized three different techniques to evaluate HPV/p16 expression in patients with anal carcinoma treated with radical RT or CRT with curative intent. We demonstrated that p16 is positive in the vast majority of patients with anal cancer, and there is good correlation of these different methods used to determine HPV tumor status. These results also support the measurement of p16 as a surrogate marker for HPV infection, using the readily available and relatively inexpensive conventional IHC technique. In a further exploratory analysis, the differential expression of p16 by AQUA identified a small subset of patients with worse prognosis on univariate but not multivariate analysis.

We recognize this is a retrospective study with some inherent weaknesses. There may be an inclusion bias as tissue samples were not available for analysis in all of the patients. Additionally, not all tests could be performed in the available samples. Technically, it is possible that HPV subtypes, in addition to the ones detected, were present but were not amplified in an amount allowing for detection. A possible lack of detection could result from inadequate specimen collection or quality. In our centre HIV testing is not routinely performed in patients who are not deemed to have sufficiently high risk factors; it was only requested in one case in this series and it was positive. We included patients who were treated with radical RT alone, in addition to radical CRT patients, if they completed curative intent treatment, which adds some heterogeneity to the population.

Overall, approximately 80% of anal SCC were positive for HPV16/p16, regardless of the method utilized. This is in agreement with other series of SCC. Varnai et al reported 86% (33/38) HPV16 positive tumors using PCR [1].

This study demonstrates the correlation between HPV16 subtype, p16 by DAB and AQUA and HPV16 CISH suggesting that testing for p16 using conventional IHC may a cost-effective way of evaluating for HPV16 status that is available in most hospital pathology departments.

We were unable to find an association between p16 expression and outcome using conventional IHC staining status. This may be related to the fact that most of the cases were HPV positive by all testing techniques, making any subset analysis by IHC score very difficult. Despite differences in the grading criteria in the literature, our results agree with the report of Ajani et al regarding p16 expression (evaluating the percentage of tumor cells with cytoplasmic staining) in 30 patients with anal cancer treated with CRT [23]. Additionally, in a cohort of 55 anal cancer patients treated with RT with or without chemotherapy, p16 staining was not significantly related to survival [24].

These results may suggest that conventional IHC grading cannot identify subgroups of anal cancer patients with different prognoses. Because most of the patients with squamous anal canal carcinoma are positive for HPV16/p16 it is possible that the classification as positive/negative is unable to identify groups with different prognosis, regardless of the method used. This is in contrast to what has been published in the head and neck cancer literature, where HPV positive versus HPV negative tumors occur in approximately equal frequency, with more favorable prognoses in patients with HPV positive tumors. In our study, automated quantitative scoring using AQUA was the only technique to identify a small subset of patients with a worse prognosis by p16 expression status, on univariate analysis. This analysis is potentially better able to quantify the expression of p16, versus conventional IHC scoring with its more limited staining quantification scale. Interesting, contrary to what has been reported in the head and neck cancer literature, our analysis suggests very high p16 status (via AQUA) was associated with worse PFS and OS, although not significant on multivariate analyses. This agrees with the results of Brambilla et al [25], who reported the association of positive p16 (evaluated by conventional IHC) with shorter survival in patients with non-small cell carcinoma of the lung. However, a series of 47 patients with anal carcinoma treated with CRT using IHC to determine p16 expression reported an association of p16 positivity with better 4-year PFS (52.5% vs. 25.0%, p = 0.01) on univariate analysis. In this study, 93.5% of HPV16 positive tumors by PCR were p16 positive but 10/16 HPV16-negative patients had p16 positive by IHC [26]. Differences in scoring and definitions of p16 positivity may explain the discordant result compared to our series and preclude direct comparisons.

To our knowledge, this is the first study to report the association of p16 determined by AQUA with outcome in patients with anal SCC. This was an exploratory analysis of an alternative method to evaluate p16 expression in a small group of patients and further evaluation of this method in an alternate dataset would be required to confirm whether very high AQUA score can distinguish patients at increased risk of treatment failure. Additional analyses will be required to determine the molecular pathway aberration(s) resulting in very high p16 status in patients with anal cancer, and the mechanisms of treatment resistance.
